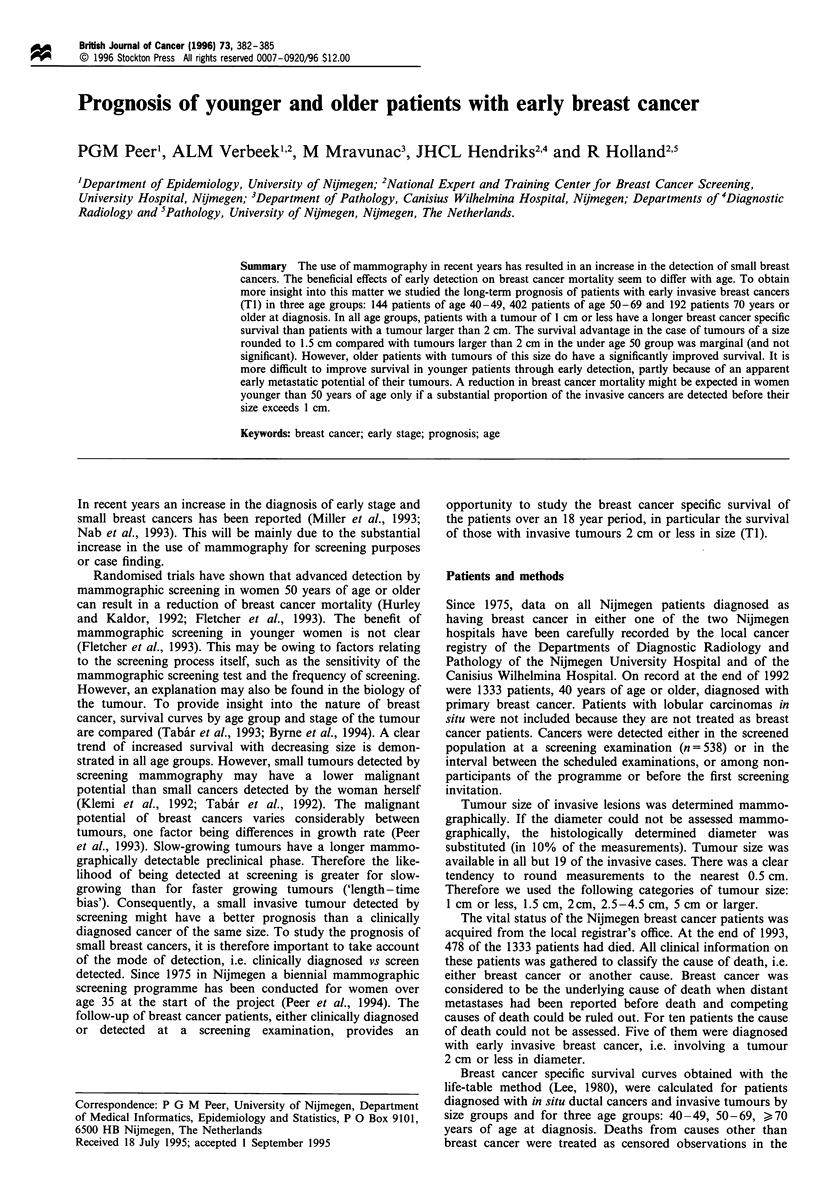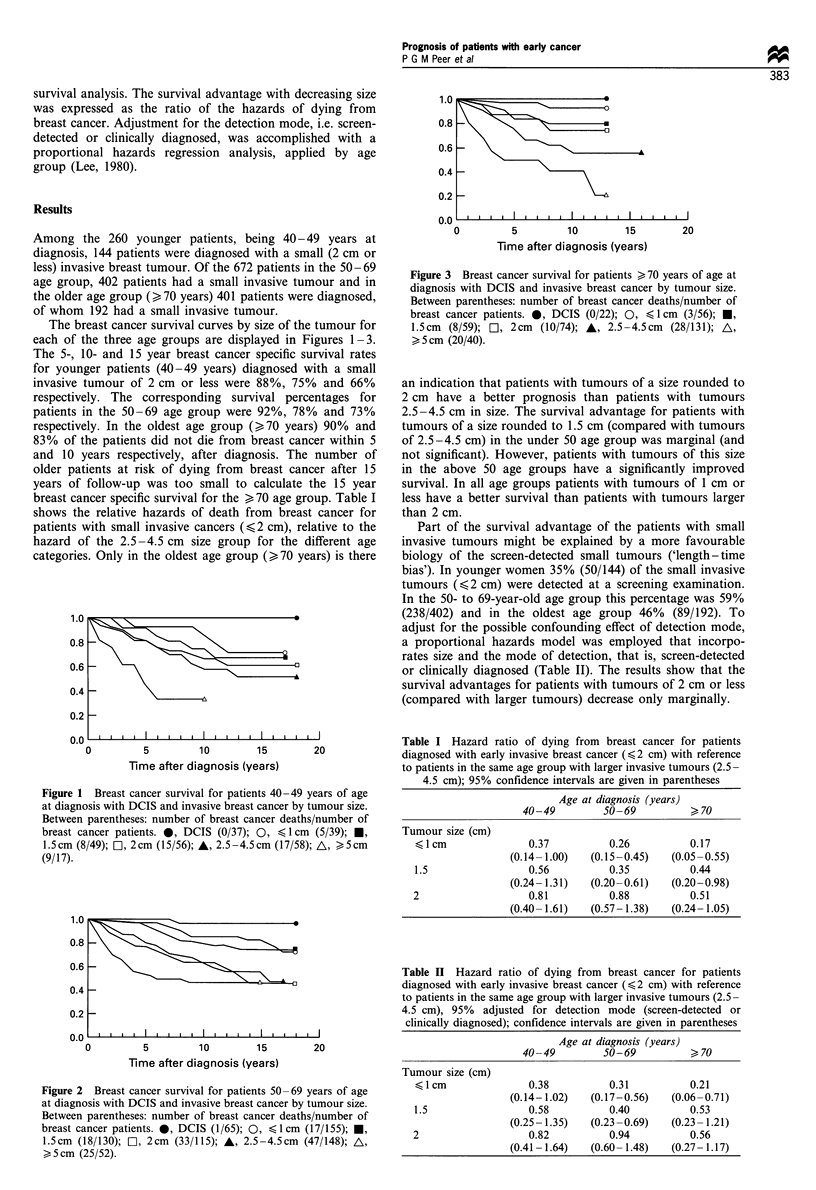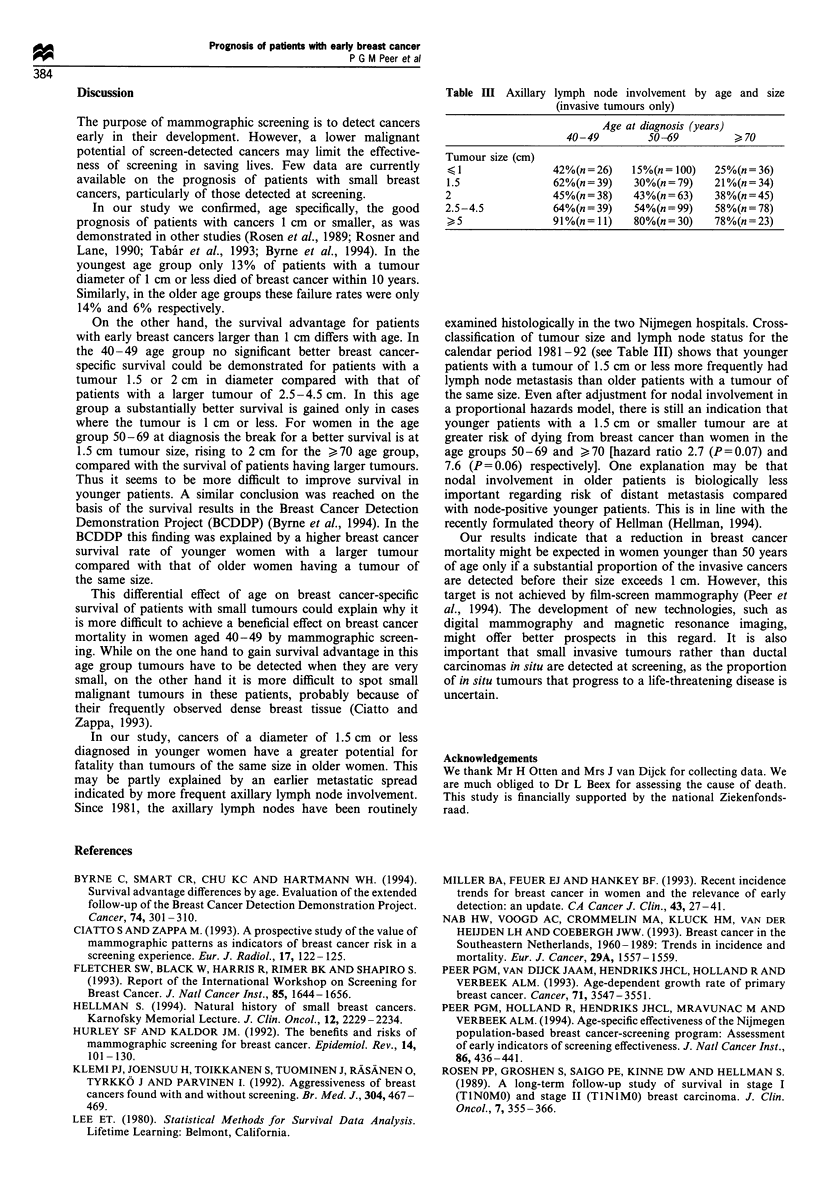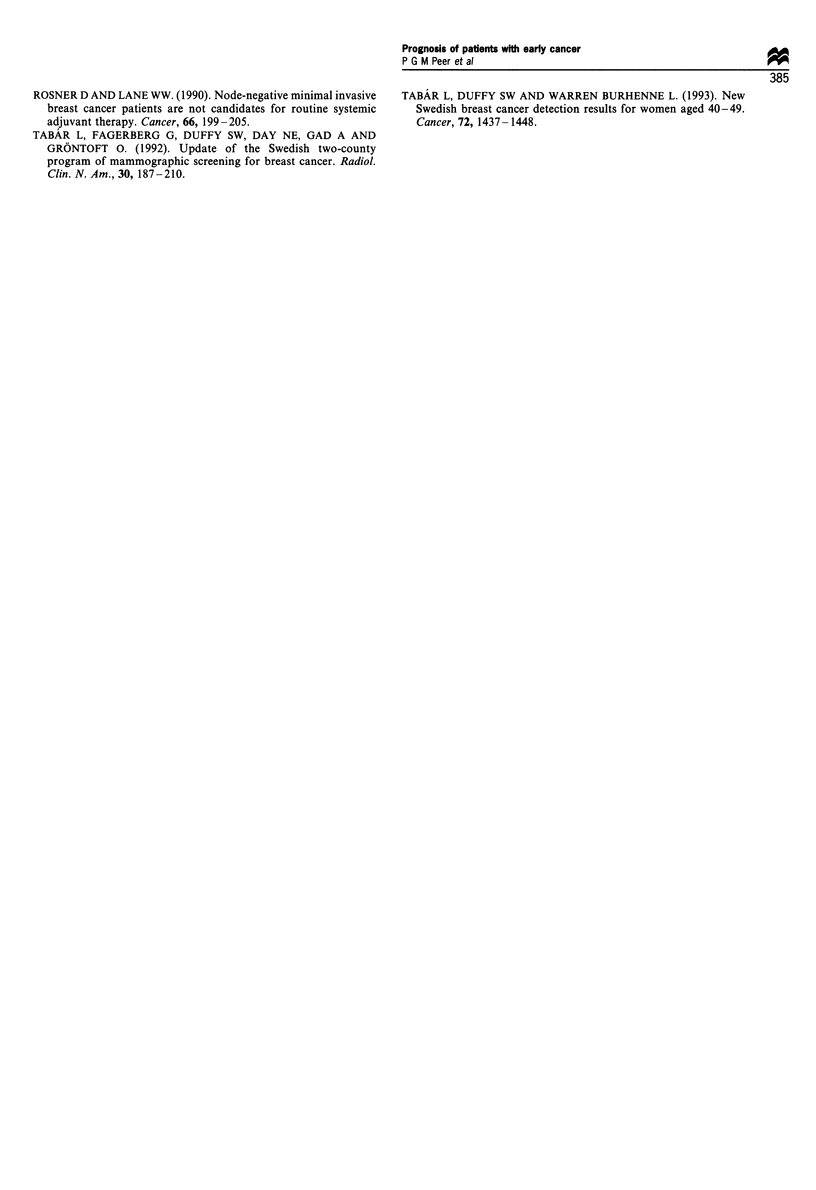# Prognosis of younger and older patients with early breast cancer.

**DOI:** 10.1038/bjc.1996.65

**Published:** 1996-02

**Authors:** P. G. Peer, A. L. Verbeek, M. Mravunac, J. H. Hendriks, R. Holland

**Affiliations:** Department of Epidemiology, University of Nijmegen, The Netherlands.

## Abstract

The use of mammography in recent years has resulted in an increase in the detection of small breast cancers. The beneficial effects of early detection on breast cancer mortality seem to differ with age. To obtain more insight into this matter we studied the long-term prognosis of patients with early invasive breast cancers (T1) in three age groups: 144 patients of age 40-49, 402 patients of age 50-69 and 192 patients 70 years or older at diagnosis. In all age groups, patients with a tumour of 1 cm or less have a longer breast cancer specific survival than patients with a tumour larger than 2 cm. The survival advantage in the case of tumours of a size rounded to 1.5 cm compared with tumours larger than 2 cm in the under age 50 group was marginal (and not significant). However, older patients with tumours of this size do have a significantly improved survival. It is more difficult to improve survival in younger patients through early detection, partly because of an apparent early metastatic potential of their tumours. A reduction in breast cancer mortality might be expected in women younger than 50 years of age only if a substantial proportion of the invasive cancers are detected before their size exceeds 1 cm.


British Journal of Cancer (1996) 73, 382-385
© 1996 Stockton Press All rights reserved 0007-0920/96 $12.00

### Prognosis of younger and older patients with early breast cancer

PGM Peer[1], ALM Verbeek[1,2], M Mravunac[3], JHCL Hendriks[2,4] and R Holland[2,5]

[1]*Department of Epidemiology, University of Nijmegen;* [2]*National Expert and Training Center for Breast Cancer Screening, University Hospital, Nijmegen;* [3]*Department of Pathology, Canisius Wilhelmina Hospital, Nijmegen; Departments of* [4]*Diagnostic Radiology and* [5]*Pathology, University of Nijmegen, Nijmegen, The Netherlands.*

**Summary** The use of mammography in recent years has resulted in an increase in the detection of small breast cancers. The beneficial effects of early detection on breast cancer mortality seem to differ with age. To obtain more insight into this matter we studied the long-term prognosis of patients with early invasive breast cancers (T1) in three age groups: 144 patients of age 40-49, 402 patients of age 50-69 and 192 patients 70 years or older at diagnosis. In all age groups, patients with a tumour of 1 cm or less have a longer breast cancer specific survival than patients with a tumour larger than 2 cm. The survival advantage in the case of tumours of a size rounded to 1.5 cm compared with tumours larger than 2 cm in the under age 50 group was marginal (and not significant). However, older patients with tumours of this size do have a significantly improved survival. It is more difficult to improve survival in younger patients through early detection, partly because of an apparent early metastatic potential of their tumours. A reduction in breast cancer mortality might be expected in women younger than 50 years of age only if a substantial proportion of the invasive cancers are detected before their size exceeds 1 cm.

**Keywords:** breast cancer; early stage; prognosis; age

In recent years an increase in the diagnosis of early stage and small breast cancers has been reported (Miller *et al.*, 1993; Nab *et al.*, 1993). This will be mainly due to the substantial increase in the use of mammography for screening purposes or case finding.

Randomised trials have shown that advanced detection by mammographic screening in women 50 years of age or older can result in a reduction of breast cancer mortality (Hurley and Kaldor, 1992; Fletcher *et al.*, 1993). The benefit of mammographic screening in younger women is not clear (Fletcher *et al.*, 1993). This may be owing to factors relating to the screening process itself, such as the sensitivity of the mammographic screening test and the frequency of screening. However, an explanation may also be found in the biology of the tumour. To provide insight into the nature of breast cancer, survival curves by age group and stage of the tumour are compared (Tabár *et al.*, 1993; Byrne *et al.*, 1994). A clear trend of increased survival with decreasing size is demonstrated in all age groups. However, small tumours detected by screening mammography may have a lower malignant potential than small cancers detected by the woman herself (Klemi *et al.*, 1992; Tabár *et al.*, 1992). The malignant potential of breast cancers varies considerably between tumours, one factor being differences in growth rate (Peer *et al.*, 1993). Slow-growing tumours have a longer mammographically detectable preclinical phase. Therefore the likelihood of being detected at screening is greater for slow-growing than for faster growing tumours ('length-time bias'). Consequently, a small invasive tumour detected by screening might have a better prognosis than a clinically diagnosed cancer of the same size. To study the prognosis of small breast cancers, it is therefore important to take account of the mode of detection, i.e. clinically diagnosed *vs* screen detected. Since 1975 in Nijmegen a biennial mammographic screening programme has been conducted for women over age 35 at the start of the project (Peer *et al.*, 1994). The follow-up of breast cancer patients, either clinically diagnosed or detected at a screening examination, provides an opportunity to study the breast cancer specific survival of the patients over an 18 year period, in particular the survival of those with invasive tumours 2 cm or less in size (T1).

### Patients and methods

Since 1975, data on all Nijmegen patients diagnosed as having breast cancer in either one of the two Nijmegen hospitals have been carefully recorded by the local cancer registry of the Departments of Diagnostic Radiology and Pathology of the Nijmegen University Hospital and of the Canisius Wilhelmina Hospital. On record at the end of 1992 were 1333 patients, 40 years of age or older, diagnosed with primary breast cancer. Patients with lobular carcinomas *in situ* were not included because they are not treated as breast cancer patients. Cancers were detected either in the screened population at a screening examination ($n=538$) or in the interval between the scheduled examinations, or among non-participants of the programme or before the first screening invitation.

Tumour size of invasive lesions was determined mammographically. If the diameter could not be assessed mammographically, the histologically determined diameter was substituted (in 10% of the measurements). Tumour size was available in all but 19 of the invasive cases. There was a clear tendency to round measurements to the nearest 0.5 cm. Therefore we used the following categories of tumour size: 1 cm or less, 1.5 cm, 2 cm, 2.5-4.5 cm, 5 cm or larger.

The vital status of the Nijmegen breast cancer patients was acquired from the local registrar's office. At the end of 1993, 478 of the 1333 patients had died. All clinical information on these patients was gathered to classify the cause of death, i.e. either breast cancer or another cause. Breast cancer was considered to be the underlying cause of death when distant metastases had been reported before death and competing causes of death could be ruled out. For ten patients the cause of death could not be assessed. Five of them were diagnosed with early invasive breast cancer, i.e. involving a tumour 2 cm or less in diameter.

Breast cancer specific survival curves obtained with the life-table method (Lee, 1980), were calculated for patients diagnosed with *in situ* ductal cancers and invasive tumours by size groups and for three age groups: 40-49, 50-69, ≥70 years of age at diagnosis. Deaths from causes other than breast cancer were treated as censored observations in the

Correspondence: P G M Peer, University of Nijmegen, Department of Medical Informatics, Epidemiology and Statistics, P O Box 9101, 6500 HB Nijmegen, The Netherlands
Received 18 July 1995; accepted 1 September 1995

survival analysis. The survival advantage with decreasing size was expressed as the ratio of the hazards of dying from breast cancer. Adjustment for the detection mode, i.e. screen-detected or clinically diagnosed, was accomplished with a proportional hazards regression analysis, applied by age group (Lee, 1980).

### Results

Among the 260 younger patients, being 40–49 years at diagnosis, 144 patients were diagnosed with a small (2 cm or less) invasive breast tumour. Of the 672 patients in the 50–69 age group, 402 patients had a small invasive tumour and in the older age group (⩾70 years) 401 patients were diagnosed, of whom 192 had a small invasive tumour.

The breast cancer survival curves by size of the tumour for each of the three age groups are displayed in Figures 1–3. The 5-, 10- and 15 year breast cancer specific survival rates for younger patients (40–49 years) diagnosed with a small invasive tumour of 2 cm or less were 88%, 75% and 66% respectively. The corresponding survival percentages for patients in the 50–69 age group were 92%, 78% and 73% respectively. In the oldest age group (⩾70 years) 90% and 83% of the patients did not die from breast cancer within 5 and 10 years respectively, after diagnosis. The number of older patients at risk of dying from breast cancer after 15 years of follow-up was too small to calculate the 15 year breast cancer specific survival for the ⩾70 age group. Table I shows the relative hazards of death from breast cancer for patients with small invasive cancers (⩽2 cm), relative to the hazard of the 2.5–4.5 cm size group for the different age categories. Only in the oldest age group (⩾70 years) is there

**Figure 1** Breast cancer survival for patients 40–49 years of age at diagnosis with DCIS and invasive breast cancer by tumour size. Between parentheses: number of breast cancer deaths/number of breast cancer patients. ●, DCIS (0/37); ○, ⩽1 cm (5/39); ■, 1.5 cm (8/49); □, 2 cm (15/56); ▲, 2.5–4.5 cm (17/58); △, ⩾5 cm (9/17).

**Figure 2** Breast cancer survival for patients 50–69 years of age at diagnosis with DCIS and invasive breast cancer by tumour size. Between parentheses: number of breast cancer deaths/number of breast cancer patients. ●, DCIS (1/65); ○, ⩽1 cm (17/155); ■, 1.5 cm (18/130); □, 2 cm (33/115); ▲, 2.5–4.5 cm (47/148); △, ⩾5 cm (25/52).

**Figure 3** Breast cancer survival for patients ⩾70 years of age at diagnosis with DCIS and invasive breast cancer by tumour size. Between parentheses: number of breast cancer deaths/number of breast cancer patients. ●, DCIS (0/22); ○, ⩽1 cm (3/56); ■, 1.5 cm (8/59); □, 2 cm (10/74); ▲, 2.5–4.5 cm (28/131); △, ⩾5 cm (20/40).

an indication that patients with tumours of a size rounded to 2 cm have a better prognosis than patients with tumours 2.5–4.5 cm in size. The survival advantage for patients with tumours of a size rounded to 1.5 cm (compared with tumours of 2.5–4.5 cm) in the under 50 age group was marginal (and not significant). However, patients with tumours of this size in the above 50 age groups have a significantly improved survival. In all age groups patients with tumours of 1 cm or less have a better survival than patients with tumours larger than 2 cm.

Part of the survival advantage of the patients with small invasive tumours might be explained by a more favourable biology of the screen-detected small tumours (‘length–time bias’). In younger women 35% (50/144) of the small invasive tumours (⩽2 cm) were detected at a screening examination. In the 50- to 69-year-old age group this percentage was 59% (238/402) and in the oldest age group 46% (89/192). To adjust for the possible confounding effect of detection mode, a proportional hazards model was employed that incorporates size and the mode of detection, that is, screen-detected or clinically diagnosed (Table II). The results show that the survival advantages for patients with tumours of 2 cm or less (compared with larger tumours) decrease only marginally.

**Table I** Hazard ratio of dying from breast cancer for patients diagnosed with early invasive breast cancer (⩽2 cm) with reference to patients in the same age group with larger invasive tumours (2.5–4.5 cm); 95% confidence intervals are given in parentheses

| | *Age at diagnosis (years)* | | |
|---|---|---|---|
| | *40–49* | *50–69* | *⩾70* |
| Tumour size (cm) | | | |
| ⩽1 cm | 0.37 | 0.26 | 0.17 |
| | (0.14–1.00) | (0.15–0.45) | (0.05–0.55) |
| 1.5 | 0.56 | 0.35 | 0.44 |
| | (0.24–1.31) | (0.20–0.61) | (0.20–0.98) |
| 2 | 0.81 | 0.88 | 0.51 |
| | (0.40–1.61) | (0.57–1.38) | (0.24–1.05) |

**Table II** Hazard ratio of dying from breast cancer for patients diagnosed with early invasive breast cancer (⩽2 cm) with reference to patients in the same age group with larger invasive tumours (2.5–4.5 cm), 95% adjusted for detection mode (screen-detected or clinically diagnosed); confidence intervals are given in parentheses

| | *Age at diagnosis (years)* | | |
|---|---|---|---|
| | *40–49* | *50–69* | *⩾70* |
| Tumour size (cm) | | | |
| ⩽1 cm | 0.38 | 0.31 | 0.21 |
| | (0.14–1.02) | (0.17–0.56) | (0.06–0.71) |
| 1.5 | 0.58 | 0.40 | 0.53 |
| | (0.25–1.35) | (0.23–0.69) | (0.23–1.21) |
| 2 | 0.82 | 0.94 | 0.56 |
| | (0.41–1.64) | (0.60–1.48) | (0.27–1.17) |

### Discussion

The purpose of mammographic screening is to detect cancers early in their development. However, a lower malignant potential of screen-detected cancers may limit the effectiveness of screening in saving lives. Few data are currently available on the prognosis of patients with small breast cancers, particularly of those detected at screening.

In our study we confirmed, age specifically, the good prognosis of patients with cancers 1 cm or smaller, as was demonstrated in other studies (Rosen *et al.*, 1989; Rosner and Lane, 1990; Tabár *et al.*, 1993; Byrne *et al.*, 1994). In the youngest age group only 13% of patients with a tumour diameter of 1 cm or less died of breast cancer within 10 years. Similarly, in the older age groups these failure rates were only 14% and 6% respectively.

On the other hand, the survival advantage for patients with early breast cancers larger than 1 cm differs with age. In the 40–49 age group no significant better breast cancer-specific survival could be demonstrated for patients with a tumour 1.5 or 2 cm in diameter compared with that of patients with a larger tumour of 2.5–4.5 cm. In this age group a substantially better survival is gained only in cases where the tumour is 1 cm or less. For women in the age group 50–69 at diagnosis the break for a better survival is at 1.5 cm tumour size, rising to 2 cm for the ⩾70 age group, compared with the survival of patients having larger tumours. Thus it seems to be more difficult to improve survival in younger patients. A similar conclusion was reached on the basis of the survival results in the Breast Cancer Detection Demonstration Project (BCDDP) (Byrne *et al.*, 1994). In the BCDDP this finding was explained by a higher breast cancer survival rate of younger women with a larger tumour compared with that of older women having a tumour of the same size.

This differential effect of age on breast cancer-specific survival of patients with small tumours could explain why it is more difficult to achieve a beneficial effect on breast cancer mortality in women aged 40–49 by mammographic screening. While on the one hand to gain survival advantage in this age group tumours have to be detected when they are very small, on the other hand it is more difficult to spot small malignant tumours in these patients, probably because of their frequently observed dense breast tissue (Ciatto and Zappa, 1993).

In our study, cancers of a diameter of 1.5 cm or less diagnosed in younger women have a greater potential for fatality than tumours of the same size in older women. This may be partly explained by an earlier metastatic spread indicated by more frequent axillary lymph node involvement. Since 1981, the axillary lymph nodes have been routinely examined histologically in the two Nijmegen hospitals. Cross-classification of tumour size and lymph node status for the calendar period 1981–92 (see Table III) shows that younger patients with a tumour of 1.5 cm or less more frequently had lymph node metastasis than older patients with a tumour of the same size. Even after adjustment for nodal involvement in a proportional hazards model, there is still an indication that younger patients with a 1.5 cm or smaller tumour are at greater risk of dying from breast cancer than women in the age groups 50–69 and ⩾70 [hazard ratio 2.7 ($P=0.07$) and 7.6 ($P=0.06$) respectively]. One explanation may be that nodal involvement in older patients is biologically less important regarding risk of distant metastasis compared with node-positive younger patients. This is in line with the recently formulated theory of Hellman (Hellman, 1994).

Our results indicate that a reduction in breast cancer mortality might be expected in women younger than 50 years of age only if a substantial proportion of the invasive cancers are detected before their size exceeds 1 cm. However, this target is not achieved by film-screen mammography (Peer *et al.*, 1994). The development of new technologies, such as digital mammography and magnetic resonance imaging, might offer better prospects in this regard. It is also important that small invasive tumours rather than ductal carcinomas *in situ* are detected at screening, as the proportion of *in situ* tumours that progress to a life-threatening disease is uncertain.

**Table III** Axillary lymph node involvement by age and size (invasive tumours only)

| | *Age at diagnosis (years)* 40–49 | 50–69 | ⩾70 |
|---|---|---|---|
| Tumour size (cm) | | | |
| ⩽1 | 42%(*n*=26) | 15%(*n*=100) | 25%(*n*=36) |
| 1.5 | 62%(*n*=39) | 30%(*n*=79) | 21%(*n*=34) |
| 2 | 45%(*n*=38) | 43%(*n*=63) | 38%(*n*=45) |
| 2.5–4.5 | 64%(*n*=39) | 54%(*n*=99) | 58%(*n*=78) |
| ⩾5 | 91%(*n*=11) | 80%(*n*=30) | 78%(*n*=23) |

**Acknowledgements**
We thank Mr H Otten and Mrs J van Dijck for collecting data. We are much obliged to Dr L Beex for assessing the cause of death. This study is financially supported by the national Ziekenfondsraad.

### References

BYRNE C, SMART CR, CHU KC AND HARTMANN WH. (1994). Survival advantage differences by age. Evaluation of the extended follow-up of the Breast Cancer Detection Demonstration Project. *Cancer*, **74**, 301–310.

CIATTO S AND ZAPPA M. (1993). A prospective study of the value of mammographic patterns as indicators of breast cancer risk in a screening experience. *Eur. J. Radiol.*, **17**, 122–125.

FLETCHER SW, BLACK W, HARRIS R, RIMER BK AND SHAPIRO S. (1993). Report of the International Workshop on Screening for Breast Cancer. *J. Natl Cancer Inst.*, **85**, 1644–1656.

HELLMAN S. (1994). Natural history of small breast cancers. Karnofsky Memorial Lecture. *J. Clin. Oncol.*, **12**, 2229–2234.

HURLEY SF AND KALDOR JM. (1992). The benefits and risks of mammographic screening for breast cancer. *Epidemiol. Rev.*, **14**, 101–130.

KLEMI PJ, JOENSUU H, TOIKKANEN S, TUOMINEN J, RÄSÄNEN O, TYRKKÖ J AND PARVINEN I. (1992). Aggressiveness of breast cancers found with and without screening. *Br. Med. J.*, **304**, 467–469.

LEE ET. (1980). *Statistical Methods for Survival Data Analysis.* Lifetime Learning: Belmont, California.

MILLER BA, FEUER EJ AND HANKEY BF. (1993). Recent incidence trends for breast cancer in women and the relevance of early detection: an update. *CA Cancer J. Clin.*, **43**, 27–41.

NAB HW, VOOGD AC, CROMMELIN MA, KLUCK HM, VAN DER HEIJDEN LH AND COEBERGH JWW. (1993). Breast cancer in the Southeastern Netherlands, 1960–1989: Trends in incidence and mortality. *Eur. J. Cancer*, **29A**, 1557–1559.

PEER PGM, VAN DIJCK JAAM, HENDRIKS JHCL, HOLLAND R AND VERBEEK ALM. (1993). Age-dependent growth rate of primary breast cancer. *Cancer*, **71**, 3547–3551.

PEER PGM, HOLLAND R, HENDRIKS JHCL, MRAVUNAC M AND VERBEEK ALM. (1994). Age-specific effectiveness of the Nijmegen population-based breast cancer-screening program: Assessment of early indicators of screening effectiveness. *J. Natl Cancer Inst.*, **86**, 436–441.

ROSEN PP, GROSHEN S, SAIGO PE, KINNE DW AND HELLMAN S. (1989). A long-term follow-up study of survival in stage I (T1N0M0) and stage II (T1N1M0) breast carcinoma. *J. Clin. Oncol.*, **7**, 355–366.

ROSNER D AND LANE WW. (1990). Node-negative minimal invasive breast cancer patients are not candidates for routine systemic adjuvant therapy. *Cancer*, **66**, 199–205.

TABÁR L, FAGERBERG G, DUFFY SW, DAY NE, GAD A AND GRÖNTOFT O. (1992). Update of the Swedish two-county program of mammographic screening for breast cancer. *Radiol. Clin. N. Am.*, **30**, 187–210.

TABÁR L, DUFFY SW AND WARREN BURHENNE L. (1993). New Swedish breast cancer detection results for women aged 40–49. *Cancer*, **72**, 1437–1448.